# Chloroform in Natural Labor

**Published:** 1849-09

**Authors:** R. P. Stevens

**Affiliations:** Ceres, Pennsylvania


*Chloroform in Natural Labor.* By R. P. Stevens, M. D., of Ceres, Pennsylvania.—Case 1.—Mrs. B. ætat. 50, primipara. She had been in labour twelve hours when I was called. By examination the os was found to be dilated to the size of a two shilling piece. It had a firm fleshy feeling, and was not easily dilated. Pains were regular but very forcing. Patient of a nervous temperament, fleshy, rosy cheeks, and in high health. In four hours the os had become sufficiently dilated for the head to be distinctly felt and exploration had. The sutures were closed, and fontanelles bony. Head was large. Pains now became very forcing, attended with great effort in bearing down. At the end of six hours from my arrival the os was sufficiently dilated for the head to pass. Great efforts made for expelling the fetus, attended with excruciating pains, and the patient piercing our ears and hearts with her cry of agony. Pulse 120, sharp and spiteful Little progress made in breaking up the head, and passing the superior strait. At this stage I administered 15 drops of chloroform on a folded napkin. In the midst of one of her most agonizing cries, while all the attendants were bathed in tears of sympathy she sank back upon her back, in a sound sweet sleep, from which, after a few minutes, she awoke, exclaiming "this is bliss, this is bliss." Pulse fell from 120 to 75 or 80 beats in the minute, and was soft and easily compressed.

The administration of ten drops of cloroform every half hour kept her in the anæsthetic state, perfectly unconscious of pain or suffering three hours, until the head was broken down and had passed the superior strait and was engaged in the inferior. Voluntary effort being suspended by the action of the chloroform, I ceased administering in full and regular doses. It was only when she was exhausted by her efforts, (which were deemed necessary) that the chloroform was given in full doses, she would then enjoy a brief season of repose, the pains still progressing but unfelt, and awoke to consciousness with renewed strength and courage for her maternal task. Seven hours she was under the influence of this anæsthetic agent, with the most happy effect. The pulse never rose above 80 after the first dose. Her recovery was rapid, and without any drawback, much more rapid than I had ever seen before, after so severe and protracted labor.

The husband hastened to make the amende d'or.

Case 2.—Mrs. W., ætat. 19, primipara. Had been in labor 24 hours, a midwife being in attendance. Her cries were heard by me before reaching the house, the distance of half a mile.

On examination the left ear was found presenting, the head lying across the superior strait. The patient was making great efforts, and her cries were loud and piercing, her eye balls seeming to start from their sockets, and the veins of the forehead and temples swelling as if about to burst.

After putting the head in a favorable position I listened to her pressing cries for chloroform, I gave her 30 drops upon a folded napkin, which in the midst of one of her most distressing pains put her into a a quiet sleep, which lasted 15 minutes. On awaking she said she had had the first quiet rest in three days, and enjoyed the most pleasing dreams.

10 to 15 drops were administered at intervals of 20 minutes or half an hour, just enough to keep her sufficiently under its influence to make her pains endurable and not suspend voluntary efforts, until the last few expulsive pains, when she was put under its full influence, and she gave birth to a fine large boy, weighing 9 lbs., undressed, wholly unconscious of suffering. She expressed herself as if having been engaged in a great effort. Her recovery was as rapid as case first.

Case 3.—Mrs. ——, ætat 40, sixth child. Her pains were not regular, frequently shifting, sometimes to the head, more frequently to the stomach, and occasionally between the shoulders. I deferred the use of chloroform until the os should become fully dilated, and the pains more regular. When this had taken place her labor pangs became "*unbearable, I would kill her,*" she said; she begged for chloroform. I exhibited 10 drops which quieted her immediately. With frequent repetitions of 5 drops, she was kept in a perfect anæsthetic state until the child was born, being entirely unconscious when it passed the external orifice. Her pulse sank from 100 to 45, under the influence of the chloroform, and continued at this low point until a half hour from the birth of her babe; it then rose slowly to her healthy standard. I

remark here, that, in the complete anæsthetic stage, she was capable at each pain of voluntary effort. Her recovery was more rapid and favorable than any of her previous confinements. She had no after pains, though previously suffering severely from them, fully equal, she said, to her true labor pains. She expressed much gratitude to me for the use of a remedy which made the hours of maternity endurable, and this anticipation pleasant.

Case 4.—Mrs. S——, ætat. 22, primipara. Premature labor at the eighth month. Breech presentation and delivery. Had been in labor 18 hours, when the os was fully dilated, but the vagina, perineum and vulva still rigid. In fact, had not begun to dilate, but was constricted, hot and dry. Pulse 120 sharp and irritable. Pains severe, forcing, occurring every ten minutes, and attended with great efforts of expulsion. Her mind was anxious for her own safety, and that of the child, and dsepairing of all hope. In previous cases of the like symptoms I bled ad deliquium animi. In this I resolved to try the chloroform; and, though the patient was much opposed, I exhibited ten drops, with the happy effect of quieting all her cries, inducing sweet and refreshing sleep, and giving hope and courage. Pulse sank to 60, and was full and soft. For three hours she was kept under the gentle influence of chloroform, giving tokens of pain only by a slight movement of the corrugator muscles of the forehead. During all this time she was conscious of what was doing in her room, of the movements of all around her, could answer questions, and wished I would put her into a perfect sleep. Cloths wrung out of hot water were kept continually upon the soft parts. At the expiration of three hours the soft parts being sufficiently dilated, the dose of chloroform was reduced, sufficiently to allow of voluntary effort, (for it had interrupted all power of aiding,) and in forty minutes she was safely delivered of a living child, quite to my surprise, having been so long in labor, with this presentation.

She expressed herself as having had the most delightful dreams, the particulars of which she could have recollected, if I had given her enough. Her recovery was rapid, and unattended with any unpleasant symptom.

I remark here, that I was as much surprised at the charming power of this agent, as I have ever been when following the advice of the late Prof. Dewees, I have bled to fainting, and seen the most unpromising cases yield to this heroic remedy. The sequelæ of the one, however, is far different from the other. After the latter, we often have to contend with all the protean symptoms of impoverished blood; while in the former, fresh air rapidly renewed the vitiated blood, and in a few hours, at the farthest, no trace of the potent agency we have been using can be found.

Case 5.—Mrs. L——, ætat. 24, seeond child, premature labor at the 7th month. Breech delivery, child stillborn, appeared to have been dead some days. The history of this case is so nearly that of No. 4, that that will suffice for this.—*Ibid.*

